# Educational interventions by nurses in caregivers with their elderly patients at home

**DOI:** 10.1017/S1463423621000086

**Published:** 2021-06-07

**Authors:** María Jesús Rojas-Ocaña, Miriam Araujo-Hernández, Rocío Romero-Castillo, E. Begoña García Navarro

**Affiliations:** Departamento de Enfermería, Facultad de Enfermería, Universidad de Huelva, Spain

**Keywords:** ageing, health care at home, informal carers, nursing interventions

## Abstract

**Introduction:**

The home is the natural setting for the development of informal care. The work that nurses are required to develop in this context (the carer/the elderly dependent/the home) focuses on training and educational activities to assist these two groups, such as demonstrating care activities to help dependent seniors, instruction in self-care techniques and teaching strategies for the use of human and material resources.

**Aims:**

This article analyzes care education interventions performed by nurses, and the factors that facilitate, or limit, health care training.

**Methodological approach:**

This is a qualitative, descriptive study designed to be flexible and openly analytical in its approach to the research problem and the dynamic nature of the home environment. Triangulation of the methodological techniques and study subjects was applied.

**Results:**

Nursing interventions related to professional attitudes, such as encouraging communication and facilitating teaching; communication interventions in health education and counseling; and technical interventions aimed at improving access to health information and support for the informal carer. Lack of will, the advanced age of the carer, emotional state and work overload are factors that undermine care instruction, which if reversed, would become learning facilitators. The lack of time and resources in the home are the major limiting factors on care teaching, according to nurses. Evidence from our study suggests that care in the home is considered a key primary health care strategy, one in which nurses play a significant role.

## Introduction

The Spanish health care system’s current care model, which has been widely developed in the autonomous community of Andalusia, southern Spain, and where community nursing is practiced (Decree on Support for Families in Andalusia, 2010; IMSERSO, 2004), situates the home, the carer and the dependent elderly as a significant feature of the professional work performed by nurses.

The work that nurses are required to develop in this context (the carer/the elderly dependent/the home) focuses on training and educational activities to assist these two groups, such as demonstrating care activities to help dependent seniors, instruction in self-care techniques and teaching strategies for the use of human and material resources. This requires the health care professional to undergo an educational process that endows them with expert knowledge and skills to deal with adult learning processes, in domestic (non-academic or non-institutional) contexts and in critical or dependent situations (health problems), and how to manage the family dynamic in the home.

This phenomenon, and the identification of its underlying problems, has been widely explored (Del Rio Lozano *et al.*, 2017), likewise, the profile of dependents and carers (Rodríguez-Madrid *et al.*, 2018), and the demands of this segment of the population and their attendant problems (IMSERSO, 2004). However, there is a significant gap in knowledge in terms of the experiences of nurses in the development of the role they play in this situation, and of the strategies and difficulties observed in this process.

At present, ‘home care is one of the basic intervention strategies undertaken by health care teams in the communities they serve. The elderly and dependents are, perhaps, the part of the population most clearly in need of this essential service’, (IMSERSO, 2004).

Professional health care interventions in the home context must demonstrate a commitment to respect the client–user – this is, after all, their personal space – a global perspective of the health care that the nurse needs to apply, and knowledge of the various intervening factors. This approach will consider structural and organizational aspects regarding the user, other family members, the home environment and service systems. It also requires the professional interests of the different groups concerned to work together (García López *et al.*, 2009). Working in the home requires a systematic approach; the home is a ‘microcosm’ where personal relations, values, physical structure, etc., coalesce to influence the type of interventions that take place indoors.

Various studies (Bohórquez *et al.*, 2011; Hall *et al.*, 2011; Puchi & y Jara, 2015; Del Rio Lozano *et al.*, 2017) highlight the significance of the setting/environment, in this case, the home, in the daily lives of residents, and their autonomy and quality of life. This is especially true of senior citizens. The ageing process affects their capacity to adapt; the elderly need more time to adjust to new spaces, and they require stable, recognizable reference points, both physical and personal, in the form of familiar faces (Fernández *et al.*, 2018). Control over a known space and its elements, feeling ‘at home’, does not require any extra effort; it is an automated response and provides a sense of security. The domestic environment is familiar and inextricably linked to the resident’s personal history (Ham *et al.*, 2012).

Care in the home is the standard-bearer of our health system. It enables us to identify and know aspects and issues that no other source of information can provide; it offers a global contextualized perspective of the problem that should boost coordination between sectors for resolution. Home care encourages the elderly to remain in their own environment, and increases family members’ sense of responsibility towards them in all aspects of their health and wellbeing. It also encourages integral patient care and strengthens the individual’s decision-taking capacity in terms of their own health problems (Genet *et al.*, 2013; Rodríguez-González *et al.*, 2017).

For centuries, attention to health problems has mainly taken place in the social space (the community) where the home is the natural context for care of the sick or dependents. The feature of this natural space is human participation (Aldana-Gonzalez & y Garcia-Gomez, 2011; Bernal *et al.*, 2018), between the carer and the person in their care. This interaction makes nursing practice much more human as it enables nurse and patient to see each other as persons, with patients having the right and obligation to be the agents of their own health, not mere passive recipients of the actions formulated by the health care professional who attends them (Vabo, 2012).

It is important to emphasize that the trend in social policy is towards prioritizing care of the elderly in the community, just as it is the expressed preference of the elderly to be cared for in their own homes (Davis & y Brayne, 2015; Bayona Huguet *et al.*, 2018).

Of equal importance to instigating social policies to resolve such problems is the identification, analysis and interpretation of how nurses’ training is to be developed, and how the nurses themselves interpret it. Attention needs to focus on where difficulties arise and which strategies are best suited to optimizing professional resources (the nurse) to fulfil the objective of resolving the health care issues of senior citizens in their homes (Casey, 2013).

The research question could be: What do nurses do when working with community-dwelling older people and their carers? The main aim of the research described in this article is to identify educational interventions by nurses that improve the care and self-care processes practiced by carers with their elderly patients in the home.

## Aims

The main aim of the research described in this article is to identify educational interventions by nurses that improve the care and self-care processes practiced by carers with their elderly patients in the home.

Specific aims:

1. To identify and observe educational interventions by nurses in the home of the elderly patient.
2. To describe the factors that facilitate, or limit, the educational process in care and self- care practice, as practiced by the carer in the patient’s home, from the perspective of nursing professionals in the field.

### Methodological approach

This is a qualitative, descriptive study designed to be flexible and openly analytical in its approach to the research problem and the dynamic nature of the home environment.

The study covered the Basic Health Zones operated by the Huelva District and Coast health authority, in southern Spain. The sample consisted of 18 health care professionals working out of health care centers in the city of Huelva. The participants were selected by intentional sample using the personal network processes in operation at such centers.

Triangulation of the methodological techniques and study subjects was applied:

1. Triangulation of subjects: the points of view of those sampled were contrasted based on the type of nursing activity developed in the various zones studied: Liaison Nurse, Community Nurse and Nurse Educator.
2. Triangulation of methods: differences and similarities were contrasted in the description and assessment of the difficulties arising from the development of the nursing intervention that is the object of this research, according to each participant’s perspective (Gatha Nursing instrument), interviews and group discussions (Figure 1).
  **Figure 1.**
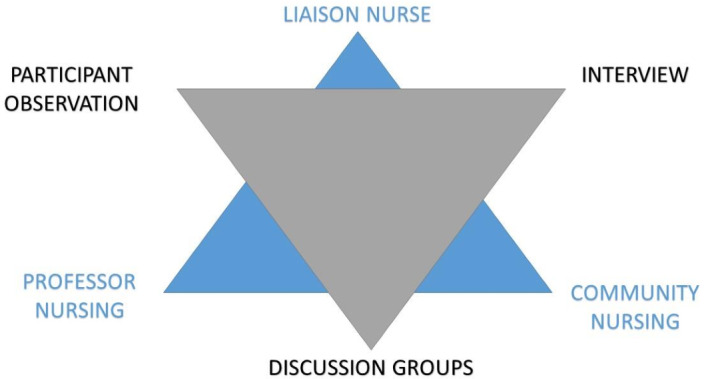
Triangulation modalities used.

### The Gatha nursing instrument

A total of 192 observations of home visits were carried out. The Gatha Nursing tool gathered 35 items on three axes: professional attitudes, communication tasks, and technical skills (Annex 1).

This instrument was designed based on work initiated by the ‘Health and Communication’ group of experts to adapt the Gatha Base (Prado *et al.*, 2003). This tool has been successfully applied in research on the communication profile of general practitioners and their training, and was initially validated by experts in 1993, 1994, and 1995. It has since been adapted to a range of media, its principal strength being the validity of its content.

Triangulation of the data concluded with eight individual interviews, four case management nurses and four community nurses, to achieve data saturation (Creswell, 1998), and a group discussion among six participants: three community nurses and two case management nurses from the Huelva District and Coast health authority, and a nursing student from the University of Huelva.

Inclusion criteria were: voluntary participation in the study, and being on active duty at the time of the interviews or group discussion.

### Data treatment: processing, analysis and interpretation

The data obtained by observation in patients’ homes using the Gatha Nursing instrument were analyzed using the Windows’ SPSS V.17 software program. This descriptive analysis enabled us to identify, using the nursing interventions classification (NIC) tool, those activities that matched the items gathered by the Gatha instrument, and the corresponding intervention. We also extracted data from the context where the nursing activity took place, and on those aspects that could facilitate, or hinder, the learning process in the home. Based on the home observations, we analyzed dimensions and categories by triangulating between the observations and the data obtained from interviews (on subjects and techniques).

Deductive coding was used based on the information from the elements observed in the home in relation to the context, and on the determining factors and interventions in the home, which were classified as dimensions, categories and subcategories.

Table 1 presents the dimensions established by the researcher for the home observations, interviews and group discussion.

**Table 1.** Collection of information in the different methods used: observation, interview, and discussion group

Dimensions	Categories		Subcategories	Observation	Interview	Discussion group
1. Context	Personal			X	X	X
	Family			X	X	X
	Environmental			X	X	X
2. Interventions	Strength		Promoting communication	X	X	X
		Cognitive Restructuring		X	X	X
		Program development		X	X	X
	Knowledge		Main caregiver support	X	X	X
			Memory training	X	X	X
			Health education	X	X	X
			Enable teaching	X	X	X
			Improving access to health information	X	X	X
	Will		Family involvement	X	X	X
			Advice	X	X	X
			Presence	X	X	X
			Active listening	X	X	X
			Setting common goals	X	X	X
			Support in decision making	X	X	X
			Empowerment of learning capacity	X	X	X
3. Determining Factors of Learning	Enablers factors	Caregivers			X	X
		Nurses		X	X	X
	Limiting factors	Caregivers			X	X
		Nurses				X
4. Professional Identity	Selfconcept				X	X
	Recognition				X	X
	Satisfaction				X	X

## Results

The results based on the methodological techniques used are as follows:

### The observations

#### Descriptive profiles

**Figure 2.**
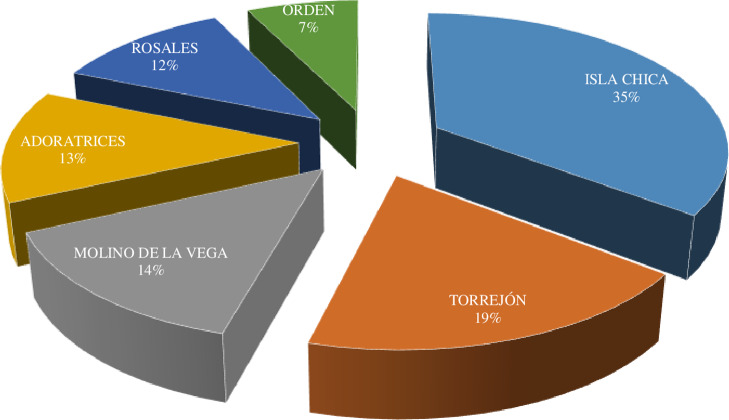
Distribution of observations by Health Centers.

The 192 visits were carried out in six health centers in the city of Huelva, Spain and were distributed as shown in Figure 2.

The number of health care professionals who undertook these home visits was 18, of whom 16.7% were male and 83.3% female. The mean age of the nurses was 48.8 with a standard deviation of 6.2. The professional profile was of 25.7 years’ experience in nursing, with a standard deviation of 5.8, and primary care experience of 15.6 years. The majority (83.3%) declared that they belonged to a professional nursing organization.

The Experience and training of nursing undergraduates in communication and clinical interviews is present in Table 2.

**Table 2.** Experience and training undergraduates nursing in communication and clinical interview

	None	Basic understanding	Sufficient theoretical and practical training
Experience in communication among nursing undergraduates	44.8%	44%	11.2%
Pre-grade training in clinical interview	27.8%	55.5%	16.7%

**Figure 3.**
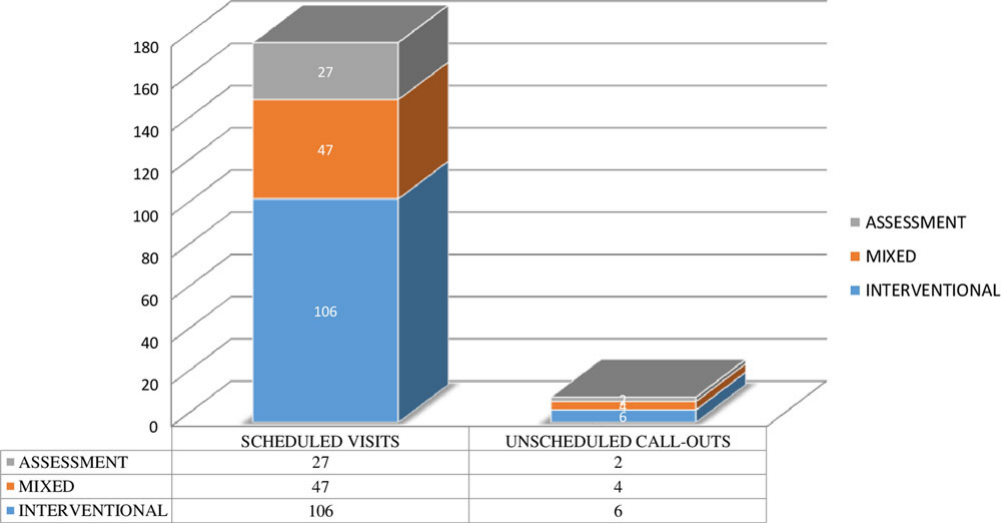
Type of inquiries and activities performed.

Within the care activity context (Gatha Nursing instrument point II), the type of attention administered (scheduled visits and unscheduled call-outs) and the type of care (interventional, mixed and assessment.) are summarized in Figure 3.

**Figure 4.**
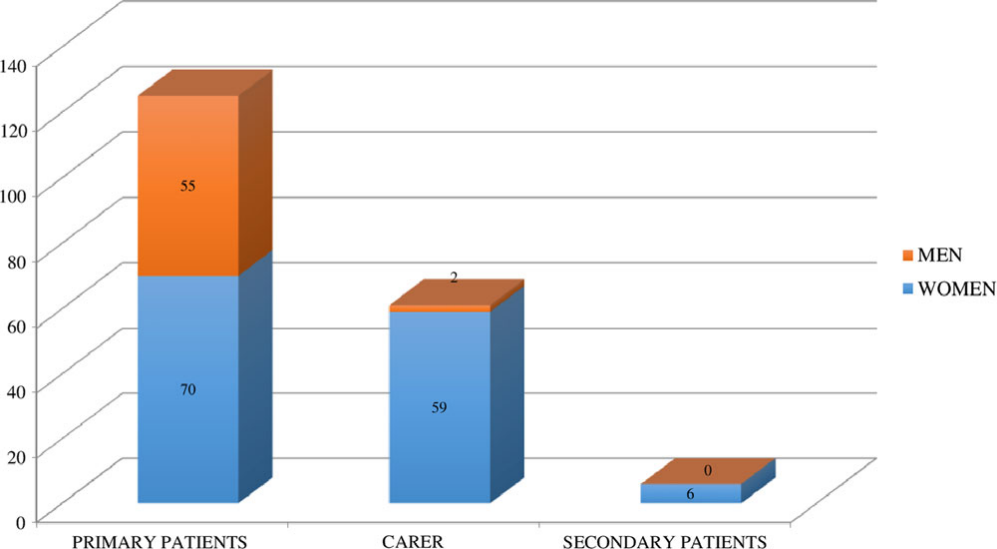
People who are the subject of nurse care. Distribution by sex.

Those who were the object of the visit were classified as primary patients, visits directed at the carer and secondary patients (to treat health problems of the carer because of their work with the patient at home). In such cases, 79.3% of those visited were women (Figure 4).

#### The Gatha instrument

Recording nurses’ educational interventions by home observation and the use of the Gatha Nursing tool was performed as follows.

An intervention by a nurse was considered completed when the item was performed in 75% or more of the observations. NIC identified those interventions that matched the items of the Gatha instrument, and the corresponding intervention (Table 3).

**Table 3.** Gatha instrument axes, items and the interventions obtained

Gatha axes	Gatha items	Interventions
Axis 1 Professional attitudes (openness, respect, closeness, understanding, empathy, security…)	1,2,3,4,6,11,12,13,14 3,4, 5,6,7,11,13 8,9,11 8,9,11,12,13 6,7,8,9 101 112,13	Fostering communication Active listening Establishing common goals Facilitating teaching Presence
Axis 2 Communication tasks: gathering information	17,19 15,16,17,18,20 19 15,17,20	Establishing common goals Health education Family involvement Counseling
Communication tasks: Establishing a care plan	21,22,23,24 25 24,25 22,23,24,25	Developing a program Support for decision taking Boosting learning capacity Counseling
Communication tasks: Monitoring the care plan	26,27 28 26,27,28	Health education Developing a program Family involvement
Axis 3 Technical skills: Gathering information	29,30 29,30	Facilitating teaching Cognitive restructuring
Technical skills: Disseminating educational content	31,32,33,34,35 31,32,34,35 31,32,34,35 31,32,34,35 31,32,34,35 31,32,34,35	Improving access to health information Facilitating teaching Health education Counseling Memory training Supporting the main carer

All these items have a compliance level of above 75%. The table shows the instrument’s axes and the Gatha items that indicate the nursing interventions.

The axes included in the Gatha Nursing instrument consist of: Axis 1. Professional Attitudes:

All 192 health care professionals (100%) begin their visit by greeting the patient on arrival in their home and addressed the patient by their name (P2).

All 192 health care professionals (100%) showed respect in their communications with the patient (P3), and smiled at some point during the session in 191 (99.5%) of the visits recorded (P4). In all cases, they looked directly at the patient when talking or listening to them (P5).

During 181 (94%) of the visits, the nurse allowed the patient to communicate, with 11 (5.7%) sessions recording no communication (not applicable) due to the patient’s pathology (P6).

On 149 (77.6%) occasions, the nurse took into consideration the patient’s opinion at all times, demonstrating a high degree of understanding (P7).

The health care professionals attended to the questions of the patient and provided clarifications in 166 (86.56%) of visits (P8).

On 179 (93.2%) occasions, we observed that the nurses treated the patient as adults without using paternalistic language (P9).

During 144 (75%) of the visits, nurses always discussed sensitive issues with those patients who manifested their fears and concerns (P10); this item was not applicable in the remainder of the sessions due to the nature of the visit, which was to perform a technical action that the patient was used to receiving.

In 183 (95.3%) of the visits, the health care professionals manifested verbal and non-verbal expressions of support and understanding with the patient (P11). All the visits were marked by nurses’ coherence in the verbal and non-verbal language used, and all said goodbye to the patient.

### Interviews

Here we describe the categories prior to the interviews, as well as those that emerge during them (context, elements that facilitate, or limit, learning in the household).

### Context

The context where the care activity is performed and its infrastructures are an important point of analysis in this study, likewise the characteristics, elements and actors in this situation. The home must be seen as a ‘microsystem’ where personal relationships, values, physical structure, etc., interact simultaneously to influence the type of intervention that takes place within. To better understand how the carers learn to carry out care activities, it is important to know the personal characteristics of the carer, their skills and capabilities, the context in which they administer the care and the resources they can count on.

The home is the space where all the human responses involving the patient and carer take place. The action of providing care to the patient is only possible if the nurse can apply the communication skills acquired to identify and characterize (diagnostic judgement phase) the general situation of the person, the health problems they present and how these problems are affected by social and psychological factors. The health care professional also needs to know how the person identifies their problem and how they feel about it, the type of mechanisms (human responses) to put into operation, and the extent to which these mechanisms function within the person’s own process. We must also consider the individual characteristics of the person, prior shared history of carer and patient, and the values and beliefs that prevail in the context, where the nurse is a guest and where they must deliver health care attention to the patient.

Three categories were used in the analysis of the dimension of context:

- Personal: the extent of the contribution of the characteristics of the carer in the home, which can involve their state of mind, level of education, willingness, physical circumstances, etc.
- Family: this aspect broadens the context by involving the social networks of each individual within the household, drawing on resources, support and family relationships.
- Environmental: this extends the notion of physical context by encompassing variables such as the household’s state of cleanliness, lighting, noise, the setting in general, etc.

Table 4 presents a summary of the segments found and grouped in categories.

**Table 4.** Categories found in the analysis of the context

Categories	Thematic core	Comments
1. Personal	1.1 Predisposition	1.1.1 ‘Some people don’t know how to read or write, and their socio-economic status is low but they are very willing for you to tell them what they can do to learn about care work.’ Liaison Nurse 3, segments 14–14.
	1.2 Illiteracy	1.2.1 ‘It is clear that people with a low level of education normally have more strength and will than those with a better education, because they have a different set of life expectations’ (Liaison Nurse 1, segments 16–16).
	1.3 Motivation and will	1.3.1 ‘Evidently, on many occasions carers have the knowledge but not the sufficient strength to put it into practice; their will in most cases has been undermined as a result of the deteriorating relationship with the person they are caring for, and this is a big influence on the care process.’ (Liaison Nurse 1, segments 4–4). 1.3.2 ‘Carers are often people of more or less the same age as the person they are taking care of, but they may be slightly better in health. This is also a problem.’ (Community Nurse 3, segments 4–4).
	1.4 Emotional burden	1.4.1 ‘Carers are always willing to collaborate, and always try to care for the patient as best they can because they know you are going to help them. Sometimes they might have a bad day, like all of us, or they cry in front of you (“I’m having a bad day. I can’t do this anymore…”).’ Community Nurse 3, segments 24–24).
2. Family	2.1 Social situation	2.1.1 ‘It is complicated for these people. You know that there are things they are not going to be able to do. Their social situation is what it is, and you leave with a feeling of impotence, because you say to yourself, “what else can I do?”’ (Liaison Nurse 3, segments 21–21).
	2.2 Family Ties	2.2.1 “The family is very important when caring for the patient, because normally wives and daughters are much more effective when there are other relatives on hand.’ (Liaison Nurse 2, segments 8–8)
	2.3 Carer–Patient Relationship	2.3.1 ‘The first thing I observe is the condition of the patient, then the relationship between patient and carer, and the state of the home, and what I can use here to do my job in the home. The resources available to them in the home’ (Community Nurse 1, segments 6–8).
	2.4 Social Support	2.4.1 ‘The first thing I observe is the condition of the patient, then the relationship between patient and carer, and the state of the home, and what I can use here to do my job in the home. The resources available to them in the home…how willing the carer is to collaborate; the first impression tells you a lot. On that first visit, you notice how the carer welcomes you into the home.’(Community Nurse 1, segments 6–8).
3. Environment	3.1 State of the Home	3.1.1 ‘While you are talking to them, you are also observing everything. While I am there, I get an impression of what the situation is. You get a good look at the home and the state it is in.’ (Liaison 3, segments 69–69). 3.1.2 ‘When you arrive, you look at the room, to see if it is tidy, with all the medication there, towels and nappies in place. If there are things they don’t know, I teach them. I might tell them to do it like this so that when I come to do the cure, I prefer it that way…and I bring them a box of stuff.’ Community Nurse 1, segments 10–10).
	3.2 Acceptance of the Role of Carer	‘But you see clearly that they are willing to do the work and that they are more than capable; they normally work hard and try to take good care of their patient, because older people have that mentality from the past, that they are the ones who do the care work. She is a woman and it’s her role to take care of the family, her husband, her children, and anyone else in the house. So, they naturally take on the role of the carer, and they feel responsible, and want to feel useful and do a perfect job.’ (Community Nurse 3, segments 18–18).

### Factors that determine learning

This dimension includes all elements that facilitate, or limit, the process of training by the nurse of the carer in the home.

Learning in this context is conditioned by the personal and situation factors analyzed in this article. Table 5 provides a summary of the two main blocks: factors that facilitate learning and factors that limit learning.

**Table 5.** Factors that determine learning

Categories	Thematic core	Comments
1. Enablers Factors	1.1 Will	1.1.1 ‘So, in the end, I think it still depends on what we were saying before, on the strength and will of the carer.’ (Liaison Nurse 1, segments 14–14). 1.1.2 ‘And some people are more willing than others to learn absolutely everything and help you in every way you ask them with their patient.’ (Liaison Nurse 2, segments 13–13).
	1.2 Physical Condition and Age	1.2.1 “One thing is that they don’t have any understanding of the job but they are very keen and in good shape physically, and in this case you can work well with them.’ (Liaison Nurse 3, segments 20–20).
	1.3 Family Support	1.3.1 ‘When there is less of a link, for example, when you have been caring for the patient on and off, like the daughter in law, or I don’t get on well with him, or I will go, what we say in these situations is that they usually just get on with their lives.’ (Liaison Nurse 2, segments 48–48).
2. Limiting Factors	2.1 Old Age	2.1.1 ‘Age, not in the sense of being old in years, but because old age means the skills needed to provide care are diminishing. Some of them just can’t do it any more. They haven’t got the energy. It’s impossible because some carers are 70 and over, and I sometimes don’t know who is in worse health, the carer or the one being cared for!’ (Liaison Nurse 3, segments 21–21). 2.1.2 ‘One woman, she really wants to help but she just can’t. She can’t change her patient’s position. She just can’t do it. She has a bad leg and uses a walking stick. Although she wants to help, it’s her age that goes against her, as in so many cases.’ (Liaison Nurse 3, segments 22–22).
	2.2 Family History	‘I’ve never got on well with him and now I have to look after him, but I’m going to do the minimum. I’ll do the basics, wash him, dress him and prepare his food but don’t ask any more of me.’ (Liaison Nurse 2, segments 11–11).

### Group discussion

The group discussion analysis yielded a new category within the dimension of factors that determine learning, defined as a subcategory – limitations on learning. This subcategory was highlighted by nurses in the group discussion, and is described in Table 6.

**Table 6.** Description of the factors that determine learning based on the techniques used

	Factors that determine learning			
	Limiting factors		Enablers factors	
Observation	Carers	Nurses	Carers	Nurses
				– Explain the reasons for the instructions given – Explain by using examples – No not use medical jargon – Check carer’s understanding of information given
INTERVIEWS			– Will – Known environment – Personal relationships in the family – Family support – Experience in providing care – Physical condition/age	
Group discussion	– Prior history of poor family relationships	– Lack of time – Experience in primary care – Lack of coordination between health care professionals – Lack of behavior modification techniques – Difficulties within management teams		

Finally, as a synthesis of the information gathered in the triangulation process involving the nurses’ perspectives and the data gathering techniques used in the study, we analyzed the data separately in the form of observation, interview, and group discussion. Table 7 shows that in the initial survey involving participant observation, the nursing interventions collated by the researcher shaped the first emerging categories that were then used in the interviews and group discussion to carry out a detailed examination of the educational interventions and activities developed in the home.

**Table 7.** Interventions based on techniques used

Techniques	Interventions		
Participant Observation	Strength	Knowledge	Will
	– Cognitive restructuring – Developing a program – Fostering communication	– Support for the main carer – Facilitate teaching – Memory training – Health education – Improve access to health information	– Family involvement – Counseling – Active listening – Establishing common goals – Presence – Support in decision taking – Boosting learning capacity
Interviews	– Developing a program	– Support for the main carer – Health education – Facilitate teaching	– Family involvement – Counseling – Active listening – Establishing common goals – Presence
Group discussion	– Cognitive restructuring – Developing a program	– Support for the main carer – Health education	– Family involvement – Counseling – Establishing common goal – Support in decision taking

## Discussion

This study aimed to determine the educational interventions developed by nurses who attend patients and liaise with carers in the home, in the Basic Health Zones operated by the Huelva District and Coast health authority, in southern Spain. The aim was also to study the factors that facilitate, or limit, this educational process.

In terms of sociodemographic features, the carer profile matches that found in other research on carers who look after the elderly (Abellán *et al.*, 2018). The carer tends to be a middle-aged woman who is responsible for domestic chores (Prado *et al.*, 2003), this person being the wife or daughter (Trivedi *et al.* 2014; Coira & y Bailon, 2014) who lives with the person they take care of; in most cases the carer is married to the patient, and has no academic qualifications (Delgado *et al.*, 2014; Del-Pino-Casado *et al.*, 2014; Abellán *et al.*, 2018). The carer is typically female, which is line with the gender stereotypes in our society (Vicente *et al.*, 2014).

### What is the nature of the context in this study?

The visits by nurses were almost entirely scheduled, by prior agreement between patient/carer and nurse. The content of the visit consisted of controlling and monitoring the patient, and was an opportunity to provide learning and support, training for the carer and in self-care techniques, among other tasks (Bernal *et al.*, 2018).

The Bernal study carried out in a home environment describes the practice of domestic care assistance, focusing on the emotional health and stress factors that affect the main carer, their spiritual wellbeing, their performance and training in care, the resistance they might display, and the execution of their technical role. The latter included administration of treatments, cures, bandages, control of vital signs, and assessment of physiological needs. The study also examined the carer’s educational role in illness prevention/promotion of good health, in preventive activities and activities promoting health education; psychosocial factors, such as the assessment of needs and social problems, and dealing with emotional problems of the patient and carer.

In a review of 33 studies on interventions involving family carers, Bustillo *et al.* (2018) found that the majority of such investigations aimed to find ways to improve carers’ emotional state and relieve the burden of work. The content and development of the interventions were not always clearly defined. Most interventions were physical–educational (Marante *et al.*, 2014; Cristancho-Lacroix *et al.*, 2015) or psychosocial (Brown *et al.*, 2015); they also carried out clinical interviews to resolve problems (Otero *et al.*, 2014) and even included phone and video phone calls (Steffen & Gant, 2016).

The interventions comprised activities developed with carers as both a resource or as a patient, with the nurse providing instruction and information. This included imparting knowledge and information on the home patient’s illness, hygiene for the bed-bound, how to change the patient’s position, adapting a diet to the patient’s needs, and safety and prevention measures (Espinoza & y Jofre, 2012; Adelman *et al.*, 2014). The literature has numerous studies centered on interventions developed to benefit carers, most of which analyze their workload, wellbeing, perceived social support, and perception of their stress levels or physical condition, among others, as in the study by Delgado *et al.* (2014).

Other personal attributes detected by nurses that are believed to intervene in the carer’s learning process are the lack of energy and motivation, difficulties arising from the family’s history, work overload and the emotional burden involved, lack of support from the rest of the family and awkward personal relationships, as highlighted by Rodriguez-Gonzalez *et al.* (2017), for example.

Conflict or lack of harmony in the family has negative consequences both for the care recipient, who might subsequently receive less support than required, and for the carer, who feels the burden of looking after the patient. Lizarraga *et al.* (2008) carried out a psychotherapeutic intervention with the aim of improving carers’ emotional state by using a cognitive-behavioral approach that applied Pearlin’s Stress Process Model (Pearlin *et al.*, 1990; Deví & Ruiz, 2002) to patient care in the home. This model uses variables such as context conditions similar to those in our study, including the carer’s sociodemographic characteristics, social and family networks, history of the relationship between patient and carer, and social support as stress factors and modulating variables.

As in our study, Bernal *et al.* (2018) found that carers’ lack of information and motivation, and lack of awareness of the situation in which they operated necessitated health care education for carers to avoid patient dependence and excessive demands on the health care system. Puchi and y Jara (2015) had similar results to ours, emphasizing the importance of communication skills when dealing with the patient and family, focusing on interventions for the care of the carer, fostering their competence in care activities, encouraging practices that strengthen the patient’s health and developing the role of health care provider.

Numerous studies have focused on general education interventions at national and international level (Rojas-Sánchez *et al.*, 2009; Guevara *et al.*, 2011; Azzolin *et al.*, 2013; Corrales & y Sánchez, 2018; Hernández Bernal *et al.*, 2018). The study on nursing communication with families by Pérez-Fernández *et al.* (2009) applied NIC to detail nursing interventions and activities, and coincided with our study in finding actions such as support for the main carer, fostering communication, active listening, and family involvement.

Azzolin *et al.* (2013) applied NIC in a study of heart failure interventions in the home; of the 11 interventions observed, 8 were shown to be effective, that is, they showed significant improvement with the performance of interventions similar to those in our context, namely, symptoms control, tolerance of the activity, and energy conservation.

Developing a Program is the cornerstone of nursing interventions, in which nurses must identify needs, prioritize objectives and describe the activities to be carried out, in other words, all steps involved in the nursing process. Puig (2009) Llobet’s thesis on care and quality of life describes how all the nursing professionals interviewed applied the Nanda Nursing Diagnosis and adhered to the nursing process in their home care visits, detecting the needs of elderly dependents and those of their carers.

Another crucial event in nurses’ home care activity is the first contact, ‘the welcome’, with the patient and family. The main objective of this first contact is to establish trust and forge a strong alliance. Pérez-Fernández *et al*. (2009) used the ‘fostering communication’ intervention in a nursing plan for welcoming the relatives of patients hospitalized in an intensive care unit, and classified it as a necessary nursing competence. Here, nurses displayed attributes similar to those found in our study, such as an open tolerant attitude, showing that they were available at all times, and maintaining positive verbal and non-verbal communication. Interventions carried out by the nurses in the study were successful although the nurses who were observed had limited formal training in communication methods, a finding which we attribute to the experience of the nurses.

The most representative interventions in the study also included counseling, active listening, establishing common goals, which we will deal with together as they are interrelated.

Various studies emphasize the importance of these aspects among nurses and home carers. (Chen *et al.* 2015), Puchi and y Sanhueza (2015) writing on health assessments carried out by nurses in the home, emphasized that the main aim of the health care professional is to gather relevant information on the factors considered essential in each case. Relevant data are a requisite for planning and performing interventions.

### Factors that determine learning

The factors that limit the ability of carers to learn good care techniques have already been discussed here. They usually relate to the carer’s lack of will and old age (Del Rio Lozano *et al.*, 2017), emotional state (López Martínez *et al.*, 2019), heavy workload (Flores *et al.*, 2012), and the social situation of the family nucleus or history of poor personal relationships, as indicated by Pearlin *et al.* (1990).

The lack of motivation and heavy workload are aspects covered by other studies (Martin-Carrasco *et al.*, 2013; Schönfeld *et al.*, 2016; Ribeiro *et al.*, 2017). In line with our study, they indicate that carers who are emotionally affected by their work find it difficult to assimilate new concepts and information.

These same attributes can be considered facilitators if reversed, that is, the carer’s will and willingness can stimulate the learning process, as well as having a strong support network and a positive attitude.

Our analysis of the factors that facilitate learning in relation to the activities performed by nurses in the home coincides with other studies (Zarit *et al.*, 2010; Crespo & y Rivas, 2015). The methodology used by nurses must adapt to the new needs of the patients; they must identify the patient’s particular characteristics and respect their experience and acquired knowledge by using language that is free of medical jargon, explaining the reason behind instructions given and encouraging feedback.

In terms of the factors that limited nurses’ ability to instruct carers and patients at home, the clear consensus among those who took part in the study was that the lack of time to attend to the patient and shortage of resources were major impediments (Otero-Lopez *et al.*, 2014; Arcos-García *et al.*, 2016).

Regarding the limitations of the study, this study was limited to the province of Huelva, which may have implications for its transferability. Likewise, the type of sampling was intentional or for convenience, which potentially introduces a possible bias in the research.

To conclude, care in the home is considered a key strategy in primary health care, and the role of nurses is crucial (Otero *et al.*, 2014; Adelman *et al.*, 2014; Otero-López *et al.*, 2014; Moral-Fernández *et al.*, 2018). Yet nurses face considerable difficulties in carrying out their duties due to the lack of socio-sanitary services and coordination of the same. Therefore, we could say that our study is confirmatory by finding multiple evidences in similar literatures.

## Conclusion

- The profile of the person who cares for the elderly at home is of a middle-aged woman, daughter or wife of the patient or dependent person, who lacks academic qualifications. They also reside with the patient and are usually married to the person they take care of. They perform personal, instrumental, and psychosocial care. The average time they have spent caring for the elderly dependent is two and a half years.
- The profile of the nursing professionals is of a married woman with an average age of 48.8 years with more than 25 years in the job. They have a low level of training in communication skills and in conducting clinical interviews.
- In the context of the home: Aspects that the nurses considered to be influential on the carer’s learning process were their lack of will or motivation, poor personal relations between family members, heavy workload and emotional strain, and lack of family support.
- Health care education was the most common intervention undertaken by nurses. This consisted of identifying the carer’s needs, and the knowledge and resources at their disposal for the nurse to be able to develop the intervention of training and skills development, based on goals and a methodology that matched the carer’s context and characteristics.
- Support for the main carer is an intervention that requires the design of innovative strategies centered on the individual needs of the carers who live in the home with the patient. Carers’ access to health education is a common intervention willingly undertaken by nurses despite the limitations imposed by lack of carer motivation, time and resources available in the home.
- Other nursing interventions found were family involvement in support of the carer, counseling, active listening, establishing common objectives, cognitive restructuring and development of a program.
- Factors that limited learning in carers were the lack of will or motivation, emotional state, work overload, a disagreeable social situation within the family, poor personal relations in the family, and the advanced age of the carer. The positive opposite of these attributes was, naturally, suited to a better atmosphere for learning.
- Factors that nurses found to facilitate learning in carers were the use of language free of medical jargon, the use of examples, explaining at all times the reasoning behind instructions given and encouraging carer feedback. Other drawbacks for carer learning in terms of nurses’ work dynamic is the shortage of time to be with the carer and patient, and inadequate training for nurses in behavior modification techniques and clinical interviews. The lack of coordination between members of the multidisciplinary team that attends to carers and their elderly patients is another hindrance found, meaning that more teamwork and better internal communication is required.

## Supplementary material

For supplementary material accompanying this paper visit http://dx.doi.org/10.1017/S1463423621000086. click here to view supplementary material
