# Supplementary material for: Educational interventions by nurses in caregivers with their elderly patients at home
Source: Prim Health Care Res Dev. 2021 Jun 7;22:e26. doi: 10.1017/S1463423621000086 (PMC8220347; doi:10.1017/S1463423621000086)

ANNEX 1: GATHA ENFERMERÍA VALIDADO

Nº de registro Observador/a:

1. CENTRO: Nombre Consulta
2. CONTEXTO DONDE SE DESARROLLA EL PROCESO DE COMUNICACIÓN

3

2

1

Tipo de atención:

A demanda Contenido:

1

Programada Derivación

3

2

Intervención Mixta Valoración

Consulta:

Inicial Seguimiento

1

2

1. PERSONA EN LA QUE SE FOCALIZA LA SESIÓN

| Rol en el proceso asistencial | 1 | Paciente primario | Sexo: | 1 | Varón | 2 | Mujer |
| --- | --- | --- | --- | --- | --- | --- | --- |
|  | 2 | Paciente secundario  → | Sexo: | 1 | Varón | 2 | Mujer |
|  | 3 | Cuidadora  → | Sexo: | 1 | Varón | 2 |  |

1. NOMBRE DEL PROFESIONAL

V. PRESENCIA DE OTRAS PERSONAS

Acompañantes

Sí Observador/a

No

1

2

1

2

VI: DURACIÓN DE LA SESIÓN EN MINUTOS:


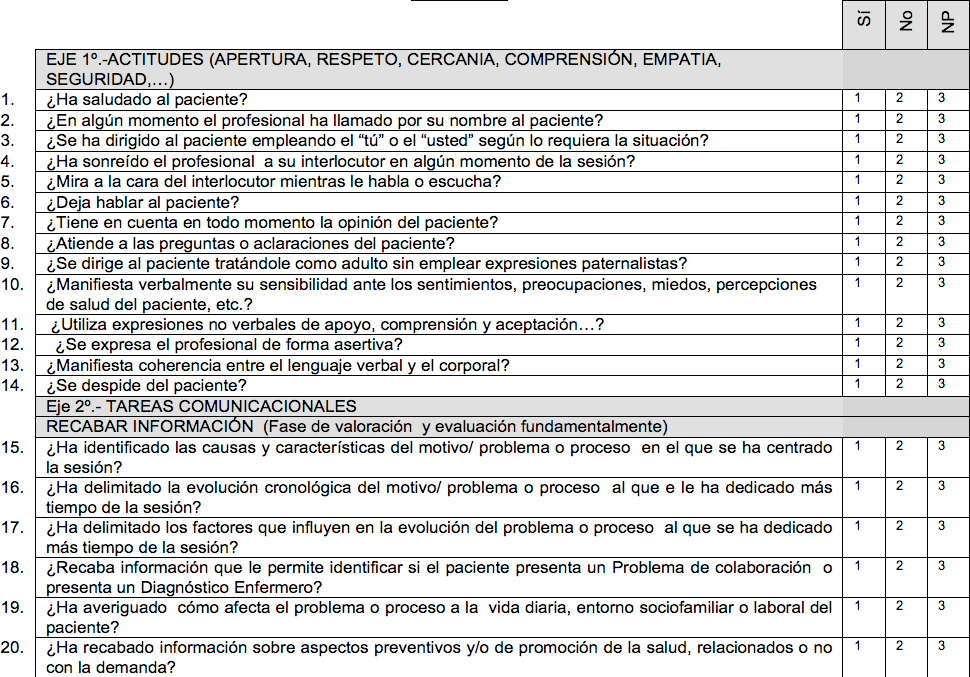


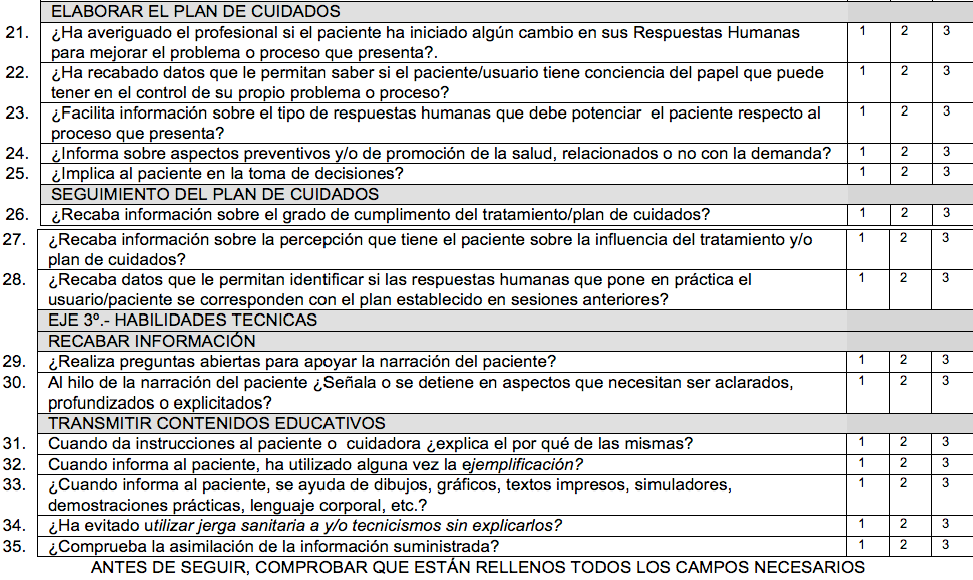

Supplement: Supplementary file 1 [file S1463423621000086sup001.docx]
